# *catena*-Poly[[[diaqua­(nitrato-κ^2^ *O*,*O*′)(2,2′:6′,2′′-terpyridine-κ^3^ *N*,*N*′,*N*′′)ytterbium(III)]-μ-cyanido-κ^2^ *N*:*C*-[dicyanido­platinum(II)]-μ-cyanido-κ^2^ *C*:*N*] acetonitrile monosolvate]

**DOI:** 10.1107/S1600536810047380

**Published:** 2010-11-20

**Authors:** Philip A. Smith, Milorad Stojanovic, Richard E. Sykora

**Affiliations:** a Department of Chemistry, University of South Alabama, Mobile, AL 36688-0002, USA

## Abstract

The title compound, {[PtYb(CN)_4_(NO_3_)(C_15_H_11_N_3_)(H_2_O)_2_]·CH_3_CN}_*n*_, was isolated from solution as a one-dimensional coordination polymer. The Yb^3+^ site has ninefold coordination with a distorted tricapped trigonal–prismatic geometry, while the Pt^II^ ion is coordinated by four cyanide groups in an almost regular square-planar geometry. *cis*-Bridging by the tetra­cyanidoplatinate(II) anions links the Yb^3+^ cations, forming chains. Additionally, each Yb^3+^ is coordinated by two water mol­ecules, one bidentate nitrate anion, and one tridentate 2,2′:6′,2′′-terpyridine mol­ecule. O—H⋯N hydrogen-bonding inter­actions are found between adjacent chains and help to consolidate the crystal packing. In addition, π–π stacking inter­actions exist between the terpyridine ligand and the two corresponding terpyridine ligands along the adjacent chain (average inter­planar distance = 3.667 Å). Moderate Pt⋯Pt inter­actions [3.5033 (4) Å] are observed in the structure.

## Related literature

For related lanthanide tetra­cyanidoplatinate structures containing 2,2′:6′,2′′- terpyridine, see: Maynard *et al.* (2008 ▶, 2010 ▶); Maynard, Smith, Ladner *et al.* (2009 ▶); Maynard, Smith & Sykora (2009 ▶). For structural and spectroscopic information on additional lanthanide tetra­cyanidoplatinates, see: Gliemann & Yersin (1985 ▶). For luminescence data on lanthanide terpyridine systems, see: Mukkala *et al.* (1995 ▶).
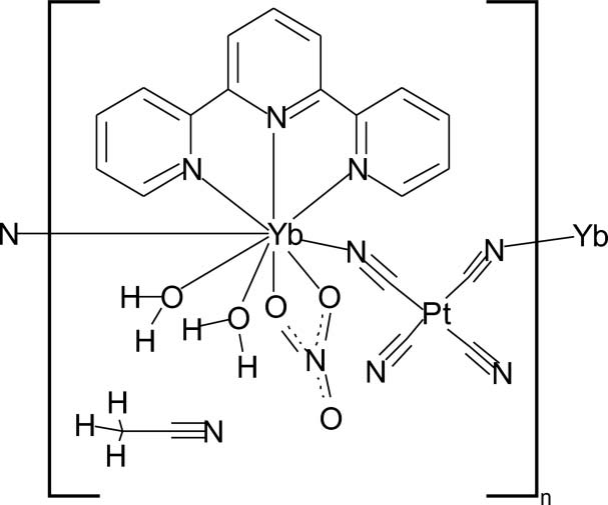


## Experimental

### 

#### Crystal data

[PtYb(CN)_4_(NO_3_)(C_15_H_11_N_3_)(H_2_O)_2_]·C_2_H_3_N
*M* *_r_* = 844.57
Triclinic, 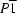

*a* = 9.0810 (3) Å
*b* = 10.1939 (3) Å
*c* = 14.4718 (6) Å
α = 79.083 (3)°
β = 72.689 (3)°
γ = 78.660 (3)°
*V* = 1241.70 (8) Å^3^
*Z* = 2
Mo *K*α radiation
μ = 9.42 mm^−1^
*T* = 290 K
0.49 × 0.31 × 0.27 mm

#### Data collection

Oxford Diffraction Xcalibur E diffractometer
Absorption correction: multi-scan (*CrysAlis PRO*; Oxford Diffraction, 2010 ▶) *T*_min_ = 0.249, *T*_max_ = 1.00
9396 measured reflections
4701 independent reflections
4049 reflections with *I* > 2σ(*I*)
*R*_int_ = 0.023

#### Refinement

*R*[*F* ^2^ > 2σ(*F* ^2^)] = 0.024
*wR*(*F* ^2^) = 0.059
*S* = 1.07
4701 reflections
336 parameters
H-atom parameters constrained
Δρ_max_ = 1.73 e Å^−3^
Δρ_min_ = −1.37 e Å^−3^

### 

Data collection: *CrysAlis PRO* (Oxford Diffraction, 2010 ▶); cell refinement: *CrysAlis PRO*; data reduction: *CrysAlis PRO*; program(s) used to solve structure: *SHELXS97* (Sheldrick, 2008 ▶); program(s) used to refine structure: *SHELXL97* (Sheldrick, 2008 ▶); molecular graphics: *OLEX2* (Dolomanov *et al.*, 2009 ▶); software used to prepare material for publication: *publCIF* (Westrip, 2010 ▶).

## Supplementary Material

Crystal structure: contains datablocks I, global. DOI: 10.1107/S1600536810047380/nc2203sup1.cif

Structure factors: contains datablocks I. DOI: 10.1107/S1600536810047380/nc2203Isup2.hkl

Additional supplementary materials:  crystallographic information; 3D view; checkCIF report

## Figures and Tables

**Table 1:** Hydrogen-bond geometry (Å, °)

*D*—H⋯*A*	*D*—H	H⋯*A*	*D*⋯*A*	*D*—H⋯*A*
O4—H4*A*⋯N9	0.85	2.07	2.868 (7)	156.5
O4—H4*B*⋯N3^i^	0.85	2.28	3.125 (6)	169.6
O5—H5*A*⋯N3^ii^	0.85	1.96	2.802 (6)	170.4
O5—H5*B*⋯N4^iii^	0.85	2.00	2.842 (6)	172.7

Symmetry codes: (i) 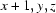; (ii) 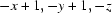; (iii) 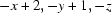.

**Fig. 1.**
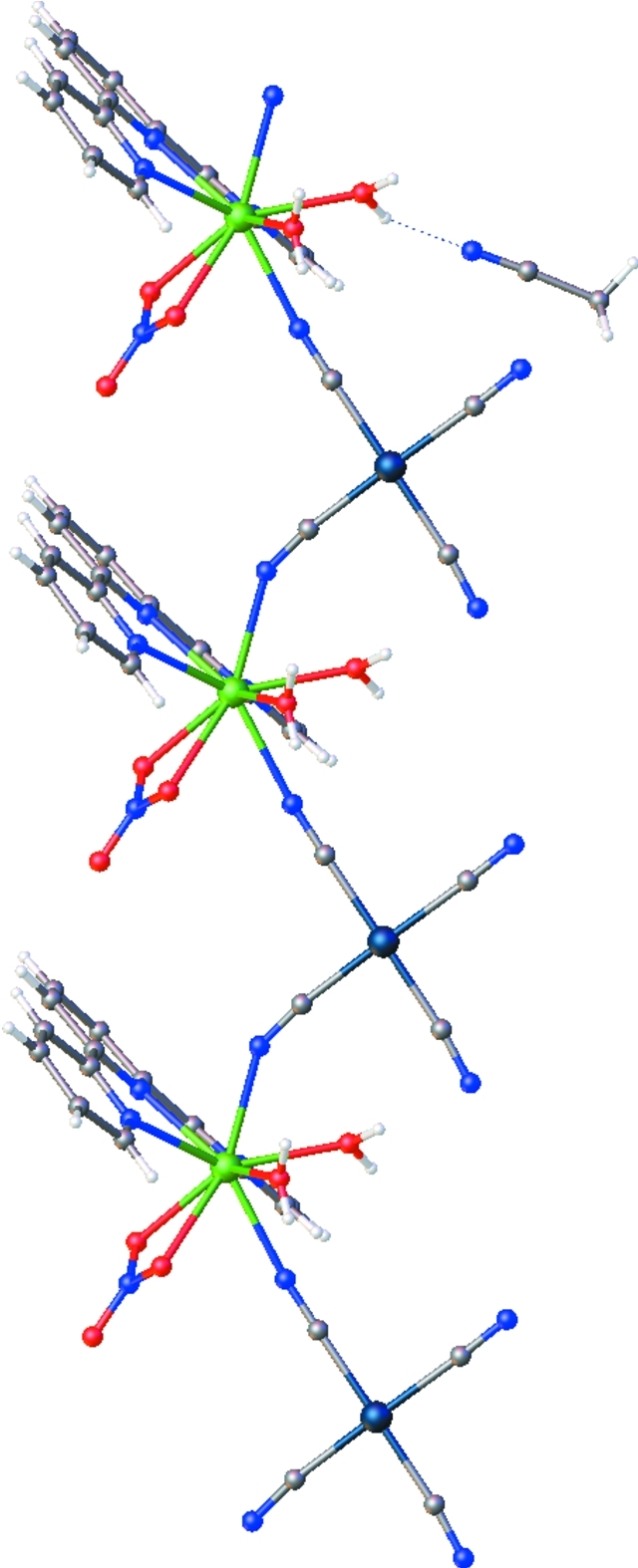
A representation of the 1—D chains that extend along the a axis in the title compound.

**Fig. 2.**
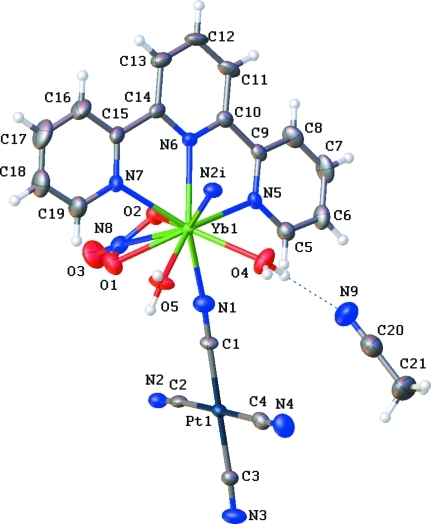
A thermal ellipsoid plot of the title compound with the atom-numbering scheme. Displacement ellipsoids for non-hydrogen atoms are drawn at the 50% probability level. H-atoms are shown as spheres of arbitrary size. Symmetry code: (i) x + 1, y, z.

**Fig. 3.**
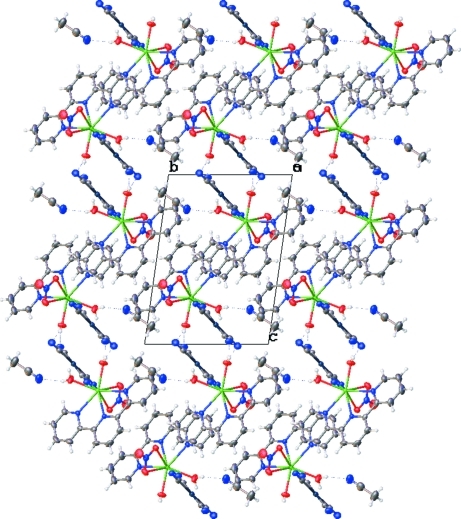
A packing diagram for the title compound viewed along the a axis, the direction parallel to the 1—D chains. H-bonding interactions are shown by the dashed lines.

## supplementary crystallographic information

### Comment

One of our research goals is to prepare systems in which weak Ln^3+^ emissions are enhanced through the use of sensitizing ligands. Recent efforts in our lab have focused on lanthanide compounds that incorporate both tetracyanidoplatinate(II) anions (TCP) and 2,2':6',2''-terpyridine (tpy), to achieve this goal (Maynard *et al.*, 2008; Maynard, Smith, Ladner *et al.*, 2009), since both of these ligands are known sensitizers for Ln^3+^ (Gliemann & Yersin, 1985; Mukkala *et al.*, 1995). We have also reported the structures of several related compounds (Maynard *et al.*, 2010; Maynard, Smith & Sykora, 2009).

The title compound is a cocrystal similar to several previously reported compounds in that it contains one-dimensional [Yb(C_15_H_11_N_3_)(H_2_O)_2_(NO_3_)Pt(CN)_4_]*_n_* chains similar to those found in Eu(C_15_H_11_N_3_)(H_2_O)_2_(NO_3_)Pt(CN)_4_].CH_3_CN (Maynard *et al.*, 2008), [Nd(C_15_H_11_N_3_)(H_2_O)_2_(NO_3_)Pt(CN)_4_].CH_3_CN.0.5C_15_H_11_N_3_ (Maynard, Smith & Sykora, 2009)), and Yb(C_15_H_11_N_3_)(H_2_O)_2_(NO_3_)Pt(CN)_4_].0.5CH_3_CN.1.5H_2_O (Maynard *et al.*, 2010). The major structural differences can be attributed to the crystallization of solvent or guest molecules between the 1—D chains. While the formulae of the title compound and Eu(C_15_H_11_N_3_)(H_2_O)_2_(NO_3_)Pt(CN)_4_].CH_3_CN appear similar, the compounds are not true isomorphs due to different packing arrangements.

The neutral, 1—D [Yb(C_15_H_11_N_3_)(H_2_O)_2_(NO_3_)(Pt(CN)_4_)]*_n_* chains in the structure of the title compound are illustrated in Figure 1 and a thermal ellipsoid plot is shown in Figure 2. The chains are formed by the linkage of the Yb^3+^ cations by *cis*-bridging TCP. The coordination of Yb1 is ninefold and is best described as a distorted [YbO_4_N_5_] tri-capped trigonal prism.

The packing diagram of the title compound along the *a* axis is shown in Figure 3. There are two dominant inter-chain features that exist in the compound: those of Pi-stacking interactions whose inter-planar distances average 3.667 Å, and Pt···Pt interactions of 3.5033 (4) Å. These former interactions are between the coordinated tpy of one chain and the tpy of the adjacent chain, overlapping one-two rings from the inversely directed tpy ligands that resideon opposite sides of the adjacent chains, thus bringing them into partial alignment. This situation is unlike that in [Eu(C_15_H_11_N_3_)(H_2_O)_2_(NO_3_)Pt(CN)_4_].CH_3_CN, where the tpy ligands are not all unidirectional along the chain, as they are in the title compound, but instead flip nearly 180° with each successive cation. The dimeric Pt···Pt interactions occur between parallel TCP ligands on adjacent chains, opposite that of the tpy Pi-stacking interactions. Additional features for the title compound include channels along the *a* axis that contain acetonitrile solvate molecules (hydrogen bound to water) and additional inter-chain O—H···N h-bonding interactions between water molcules and TCP anions of neighboring chains (Table 1).

### Experimental

Yb(NO_3_)_3_.6H_2_O (Strem, 99.9%), K_2_Pt(CN)_4_.3H_2_O (Alfa Aesar, 99.9%), and 2,2':6',2''-terpyridine (tpy) (Aldrich, 98%) were used as received without further purification. The reaction proceeded by reacting a 1:1:1 molar ratio of reactants: 1 ml of a 0.10 *M* solution of Yb(NO_3_)_3_ was mixed with 1 ml of a 0.10 *M* solution of Pt(CN)_4_^2–^ followed by layering of 1 ml of 0.10 *M* tpy. The tpy and Yb^3+^ solutions were prepared in acetonitrile, whereas the Pt(CN)_4_^2–^ solution was prepared using acetonitrile, with drop-wise addition of H_2_O until the K_2_Pt(CN)_4_.3H_2_O was completely dissolved. Slow evaporation of the solvents over a period of 1–2 weeks resulted in the crystallization of the title compound as colorless prisms in a yield of 46%.

### Refinement

Hydrogen atoms on the terpyridine ring and acetonitrile molecule were placed in calculated positions (the acetonitrile H atoms were allowed to rotate but not to tip) and allowed to ride during subsequent refinement, with *U*_iso_(H) = 1.2*U*_eq_(C) and C—H distances of 0.93 Å for the former and *U*_iso_(H) = 1.5*U*_eq_(C) and C—H distances of 0.96 Å for the latter. H-atoms contained in the water molecules were initially located in the difference map and then constrained to have O—H distances of 0.85 Å and *U*_iso_(H) = 1.5*U*_eq_(O).

### Crystal data

**Table:**

[PtYb(CN)_4_(NO_3_)(C_15_H_11_N_3_)(H_2_O)_2_]·C_2_H_3_N	*Z* = 2
*M**_r_* = 844.57	*F*(000) = 790
Triclinic, *P*1	*D*_x_ = 2.259 Mg m^−^^3^
Hall symbol: -P 1	Mo *K*α radiation, λ = 0.71073 Å
*a* = 9.0810 (3) Å	Cell parameters from 8297 reflections
*b* = 10.1939 (3) Å	θ = 3.1–25.6°
*c* = 14.4718 (6) Å	µ = 9.42 mm^−^^1^
α = 79.083 (3)°	*T* = 290 K
β = 72.689 (3)°	Prism, colorless
γ = 78.660 (3)°	0.49 × 0.31 × 0.27 mm
*V* = 1241.70 (8) Å^3^	

### Data collection

**Table:**

Oxford Diffraction Xcalibur E diffractometer	4701 independent reflections
Radiation source: fine-focus sealed tube	4049 reflections with *I* > 2σ(*I*)
graphite	*R*_int_ = 0.023
Detector resolution: 16.0514 pixels mm^-1^	θ_max_ = 25.7°, θ_min_ = 3.1°
ω scans	*h* = −7→11
Absorption correction: multi-scan (*CrysAlis PRO*; Oxford Diffraction, 2010)	*k* = −12→12
*T*_min_ = 0.249, *T*_max_ = 1.00	*l* = −16→17
9396 measured reflections	

### Refinement

**Table:**

Refinement on *F*^2^	Secondary atom site location: difference Fourier map
Least-squares matrix: full	Hydrogen site location: inferred from neighbouring sites
*R*[*F*^2^ > 2σ(*F*^2^)] = 0.024	H-atom parameters constrained
*wR*(*F*^2^) = 0.059	*w* = 1/[σ^2^(*F*_o_^2^) + (0.0345*P*)^2^] where *P* = (*F*_o_^2^ + 2*F*_c_^2^)/3
*S* = 1.07	(Δ/σ)_max_ = 0.001
4701 reflections	Δρ_max_ = 1.73 e Å^−^^3^
336 parameters	Δρ_min_ = −1.37 e Å^−^^3^
0 restraints	Extinction correction: *SHELXL97* (Sheldrick, 2008), Fc^*^=kFc[1+0.001xFc^2^λ^3^/sin(2θ)]^-1/4^
Primary atom site location: structure-invariant direct methods	Extinction coefficient: 0.00308 (18)

### Special details

**Table:**

Geometry. All e.s.d.'s (except the e.s.d. in the dihedral angle between two l.s. planes) are estimated using the full covariance matrix. The cell e.s.d.'s are taken into account individually in the estimation of e.s.d.'s in distances, angles and torsion angles; correlations between e.s.d.'s in cell parameters are only used when they are defined by crystal symmetry. An approximate (isotropic) treatment of cell e.s.d.'s is used for estimating e.s.d.'s involving l.s. planes.
Refinement. Refinement of *F*^2^ against ALL reflections. The weighted *R*-factor *wR* and goodness of fit *S* are based on *F*^2^, conventional *R*-factors *R* are based on *F*, with *F* set to zero for negative *F*^2^. The threshold expression of *F*^2^ > 2σ(*F*^2^) is used only for calculating *R*-factors(gt) *etc*. and is not relevant to the choice of reflections for refinement. *R*-factors based on *F*^2^ are statistically about twice as large as those based on *F*, and *R*- factors based on ALL data will be even larger.

### Fractional atomic coordinates and isotropic or equivalent isotropic displacement parameters (Å^2^)

**Table:**

	*x*	*y*	*z*	*U*_iso_*/*U*_eq_	
Yb1	0.92271 (2)	0.322575 (18)	0.273932 (13)	0.01623 (8)	
Pt1	0.435115 (18)	0.553063 (17)	0.115578 (12)	0.01752 (7)	
C1	0.5961 (5)	0.4697 (5)	0.1840 (3)	0.0208 (10)	
C2	0.2881 (5)	0.4280 (5)	0.1947 (3)	0.0222 (10)	
C3	0.2702 (5)	0.6376 (5)	0.0495 (3)	0.0222 (10)	
C4	0.5788 (6)	0.6826 (5)	0.0358 (4)	0.0262 (11)	
C5	0.6801 (6)	0.5675 (5)	0.3990 (4)	0.0290 (12)	
H5	0.6560	0.5923	0.3394	0.035*	
C6	0.5988 (6)	0.6420 (5)	0.4742 (4)	0.0347 (13)	
H6	0.5235	0.7156	0.4647	0.042*	
C7	0.6317 (7)	0.6050 (6)	0.5630 (4)	0.0413 (15)	
H7	0.5789	0.6526	0.6150	0.050*	
C8	0.7439 (6)	0.4963 (6)	0.5732 (4)	0.0349 (13)	
H8	0.7667	0.4691	0.6332	0.042*	
C9	0.8235 (5)	0.4269 (5)	0.4960 (3)	0.0222 (11)	
C10	0.9491 (5)	0.3122 (5)	0.5027 (3)	0.0236 (11)	
C11	1.0075 (6)	0.2769 (6)	0.5838 (4)	0.0337 (13)	
H11	0.9669	0.3254	0.6364	0.040*	
C12	1.1257 (7)	0.1696 (6)	0.5862 (4)	0.0387 (14)	
H12	1.1661	0.1452	0.6401	0.046*	
C13	1.1823 (6)	0.1004 (5)	0.5088 (4)	0.0338 (13)	
H13	1.2636	0.0288	0.5086	0.041*	
C14	1.1188 (5)	0.1363 (5)	0.4292 (4)	0.0230 (11)	
C15	1.1671 (5)	0.0578 (5)	0.3465 (4)	0.0243 (11)	
C16	1.2804 (6)	−0.0557 (5)	0.3421 (5)	0.0390 (14)	
H16	1.3341	−0.0816	0.3898	0.047*	
C17	1.3126 (7)	−0.1298 (6)	0.2663 (5)	0.0510 (17)	
H17	1.3889	−0.2057	0.2621	0.061*	
C18	1.2322 (8)	−0.0915 (6)	0.1978 (5)	0.0464 (16)	
H18	1.2510	−0.1417	0.1470	0.056*	
C19	1.1228 (7)	0.0226 (5)	0.2048 (4)	0.0364 (13)	
H19	1.0689	0.0495	0.1572	0.044*	
C20	0.7057 (9)	0.9079 (7)	0.1599 (5)	0.0537 (17)	
C21	0.6432 (11)	1.0314 (7)	0.1050 (6)	0.076 (3)	
H21A	0.7216	1.0895	0.0790	0.114*	
H21B	0.5537	1.0770	0.1478	0.114*	
H21C	0.6134	1.0085	0.0525	0.114*	
N1	0.6910 (5)	0.4234 (4)	0.2230 (3)	0.0305 (10)	
N2	0.1890 (5)	0.3662 (4)	0.2380 (3)	0.0250 (9)	
N3	0.1670 (5)	0.6804 (5)	0.0164 (3)	0.0359 (11)	
N4	0.6653 (5)	0.7528 (5)	−0.0099 (3)	0.0381 (12)	
N5	0.7917 (4)	0.4617 (4)	0.4075 (3)	0.0223 (9)	
N6	1.0054 (4)	0.2434 (4)	0.4258 (3)	0.0196 (8)	
N7	1.0901 (4)	0.0980 (4)	0.2785 (3)	0.0232 (9)	
N8	0.6955 (5)	0.1430 (4)	0.3293 (3)	0.0285 (10)	
N9	0.7471 (9)	0.8127 (6)	0.2038 (5)	0.0695 (18)	
O1	0.7876 (4)	0.1492 (4)	0.2458 (3)	0.0356 (9)	
O2	0.7216 (4)	0.2084 (3)	0.3877 (2)	0.0278 (8)	
O3	0.5860 (5)	0.0800 (4)	0.3544 (3)	0.0509 (12)	
O4	0.9449 (4)	0.5562 (4)	0.2090 (3)	0.0386 (10)	
H4A	0.8668	0.6185	0.2186	0.058*	
H4B	1.0057	0.5805	0.1537	0.058*	
O5	1.0152 (4)	0.3114 (4)	0.1110 (2)	0.0325 (9)	
H5A	0.9568	0.3053	0.0762	0.049*	
H5B	1.1114	0.2996	0.0806	0.049*	

### Atomic displacement parameters (Å^2^)

**Table:**

	*U*^11^	*U*^22^	*U*^33^	*U*^12^	*U*^13^	*U*^23^
Yb1	0.01534 (11)	0.01848 (12)	0.01563 (12)	−0.00102 (8)	−0.00609 (8)	−0.00271 (8)
Pt1	0.01570 (10)	0.01992 (11)	0.01880 (11)	−0.00175 (7)	−0.00724 (7)	−0.00400 (8)
C1	0.015 (2)	0.027 (3)	0.022 (3)	−0.0023 (19)	−0.009 (2)	−0.003 (2)
C2	0.023 (2)	0.024 (3)	0.022 (3)	0.002 (2)	−0.014 (2)	−0.003 (2)
C3	0.025 (2)	0.024 (3)	0.020 (3)	−0.005 (2)	−0.009 (2)	−0.001 (2)
C4	0.024 (2)	0.031 (3)	0.026 (3)	−0.001 (2)	−0.007 (2)	−0.012 (2)
C5	0.031 (3)	0.029 (3)	0.028 (3)	−0.004 (2)	−0.008 (2)	−0.007 (2)
C6	0.027 (3)	0.027 (3)	0.046 (4)	0.001 (2)	−0.002 (2)	−0.016 (3)
C7	0.042 (3)	0.040 (3)	0.037 (3)	−0.008 (3)	0.009 (3)	−0.024 (3)
C8	0.038 (3)	0.043 (3)	0.023 (3)	−0.010 (3)	0.000 (2)	−0.011 (2)
C9	0.024 (2)	0.024 (3)	0.020 (3)	−0.011 (2)	−0.004 (2)	−0.004 (2)
C10	0.027 (2)	0.024 (3)	0.022 (3)	−0.009 (2)	−0.007 (2)	−0.001 (2)
C11	0.046 (3)	0.044 (3)	0.016 (3)	−0.017 (3)	−0.012 (2)	−0.001 (2)
C12	0.053 (4)	0.042 (3)	0.033 (3)	−0.016 (3)	−0.032 (3)	0.010 (3)
C13	0.038 (3)	0.031 (3)	0.037 (3)	−0.004 (2)	−0.023 (3)	0.004 (3)
C14	0.024 (2)	0.019 (2)	0.029 (3)	−0.0081 (19)	−0.013 (2)	0.004 (2)
C15	0.023 (2)	0.017 (2)	0.036 (3)	−0.0053 (19)	−0.013 (2)	−0.001 (2)
C16	0.035 (3)	0.030 (3)	0.054 (4)	0.007 (2)	−0.021 (3)	−0.006 (3)
C17	0.037 (3)	0.030 (3)	0.077 (5)	0.010 (3)	−0.008 (3)	−0.014 (3)
C18	0.052 (4)	0.032 (3)	0.051 (4)	0.002 (3)	−0.004 (3)	−0.019 (3)
C19	0.047 (3)	0.029 (3)	0.032 (3)	0.001 (3)	−0.009 (3)	−0.009 (2)
C20	0.073 (5)	0.045 (4)	0.054 (4)	−0.009 (4)	−0.032 (4)	−0.011 (3)
C21	0.126 (7)	0.042 (4)	0.077 (6)	−0.023 (5)	−0.060 (5)	0.013 (4)
N1	0.026 (2)	0.038 (3)	0.028 (2)	−0.005 (2)	−0.0088 (19)	−0.002 (2)
N2	0.025 (2)	0.029 (2)	0.024 (2)	−0.0059 (18)	−0.0107 (18)	−0.0006 (19)
N3	0.033 (2)	0.049 (3)	0.029 (3)	−0.002 (2)	−0.017 (2)	−0.003 (2)
N4	0.036 (3)	0.042 (3)	0.037 (3)	−0.016 (2)	−0.003 (2)	−0.006 (2)
N5	0.027 (2)	0.020 (2)	0.021 (2)	−0.0055 (17)	−0.0051 (17)	−0.0056 (17)
N6	0.0231 (19)	0.019 (2)	0.018 (2)	−0.0045 (16)	−0.0079 (16)	−0.0003 (16)
N7	0.024 (2)	0.018 (2)	0.029 (2)	0.0002 (16)	−0.0091 (18)	−0.0072 (17)
N8	0.023 (2)	0.025 (2)	0.038 (3)	−0.0027 (18)	−0.012 (2)	−0.001 (2)
N9	0.110 (5)	0.040 (3)	0.065 (4)	0.006 (3)	−0.046 (4)	−0.005 (3)
O1	0.0312 (19)	0.041 (2)	0.038 (2)	−0.0078 (17)	−0.0063 (18)	−0.0178 (18)
O2	0.0313 (18)	0.0274 (19)	0.0269 (19)	−0.0089 (15)	−0.0072 (16)	−0.0053 (16)
O3	0.038 (2)	0.054 (3)	0.068 (3)	−0.029 (2)	−0.012 (2)	−0.008 (2)
O4	0.0292 (19)	0.025 (2)	0.048 (2)	−0.0009 (16)	0.0019 (17)	0.0051 (18)
O5	0.0223 (17)	0.058 (2)	0.0196 (18)	−0.0038 (17)	−0.0087 (15)	−0.0094 (17)

### Geometric parameters (Å, °)

**Table:**

Yb1—O5	2.272 (3)	C11—C12	1.377 (8)
Yb1—O2	2.392 (3)	C11—H11	0.9300
Yb1—N1	2.411 (4)	C12—C13	1.353 (8)
Yb1—O4	2.413 (3)	C12—H12	0.9300
Yb1—N2^i^	2.430 (4)	C13—C14	1.394 (7)
Yb1—N6	2.473 (4)	C13—H13	0.9300
Yb1—N5	2.484 (4)	C14—N6	1.350 (6)
Yb1—N7	2.488 (4)	C14—C15	1.476 (7)
Yb1—O1	2.494 (4)	C15—N7	1.330 (6)
Yb1—N8	2.863 (4)	C15—C16	1.387 (7)
Pt1—C1	1.973 (5)	C16—C17	1.377 (9)
Pt1—C2	1.981 (5)	C16—H16	0.9300
Pt1—C3	1.983 (5)	C17—C18	1.356 (9)
Pt1—C4	1.996 (5)	C17—H17	0.9300
C1—N1	1.146 (6)	C18—C19	1.371 (8)
C2—N2	1.156 (6)	C18—H18	0.9300
C3—N3	1.151 (6)	C19—N7	1.360 (7)
C4—N4	1.140 (6)	C19—H19	0.9300
C5—N5	1.341 (6)	C20—N9	1.118 (8)
C5—C6	1.386 (7)	C20—C21	1.465 (9)
C5—H5	0.9300	C21—H21A	0.9600
C6—C7	1.371 (8)	C21—H21B	0.9600
C6—H6	0.9300	C21—H21C	0.9600
C7—C8	1.368 (8)	N2—Yb1^ii^	2.430 (4)
C7—H7	0.9300	N8—O3	1.217 (5)
C8—C9	1.375 (7)	N8—O1	1.247 (5)
C8—H8	0.9300	N8—O2	1.269 (5)
C9—N5	1.363 (6)	O4—H4A	0.8500
C9—C10	1.475 (7)	O4—H4B	0.8500
C10—N6	1.345 (6)	O5—H5A	0.8499
C10—C11	1.387 (7)	O5—H5B	0.8500
			
O5—Yb1—O2	126.83 (12)	C9—C8—H8	119.6
O5—Yb1—N1	80.34 (13)	N5—C9—C8	121.5 (5)
O2—Yb1—N1	75.96 (13)	N5—C9—C10	115.8 (4)
O5—Yb1—O4	78.62 (14)	C8—C9—C10	122.7 (5)
O2—Yb1—O4	134.27 (12)	N6—C10—C11	121.3 (5)
N1—Yb1—O4	71.99 (14)	N6—C10—C9	116.7 (4)
O5—Yb1—N2^i^	77.33 (13)	C11—C10—C9	122.0 (5)
O2—Yb1—N2^i^	145.37 (13)	C12—C11—C10	119.8 (5)
N1—Yb1—N2^i^	137.41 (13)	C12—C11—H11	120.1
O4—Yb1—N2^i^	68.35 (13)	C10—C11—H11	120.1
O5—Yb1—N6	139.51 (12)	C13—C12—C11	118.9 (5)
O2—Yb1—N6	72.88 (12)	C13—C12—H12	120.6
N1—Yb1—N6	139.64 (14)	C11—C12—H12	120.6
O4—Yb1—N6	114.33 (13)	C12—C13—C14	120.0 (5)
N2^i^—Yb1—N6	73.30 (12)	C12—C13—H13	120.0
O5—Yb1—N5	148.52 (13)	C14—C13—H13	120.0
O2—Yb1—N5	71.87 (12)	N6—C14—C13	121.1 (5)
N1—Yb1—N5	80.97 (14)	N6—C14—C15	116.5 (4)
O4—Yb1—N5	71.61 (13)	C13—C14—C15	122.4 (5)
N2^i^—Yb1—N5	100.34 (13)	N7—C15—C16	121.6 (5)
N6—Yb1—N5	65.42 (13)	N7—C15—C14	116.2 (4)
O5—Yb1—N7	80.43 (13)	C16—C15—C14	122.2 (5)
O2—Yb1—N7	85.64 (12)	C17—C16—C15	119.3 (5)
N1—Yb1—N7	137.01 (14)	C17—C16—H16	120.4
O4—Yb1—N7	139.58 (12)	C15—C16—H16	120.4
N2^i^—Yb1—N7	73.55 (13)	C18—C17—C16	119.6 (5)
N6—Yb1—N7	64.99 (13)	C18—C17—H17	120.2
N5—Yb1—N7	129.60 (13)	C16—C17—H17	120.2
O5—Yb1—O1	75.44 (13)	C17—C18—C19	118.8 (6)
O2—Yb1—O1	51.64 (12)	C17—C18—H18	120.6
N1—Yb1—O1	68.10 (14)	C19—C18—H18	120.6
O4—Yb1—O1	135.18 (13)	N7—C19—C18	122.6 (6)
N2^i^—Yb1—O1	137.30 (13)	N7—C19—H19	118.7
N6—Yb1—O1	109.16 (12)	C18—C19—H19	118.7
N5—Yb1—O1	119.84 (12)	N9—C20—C21	177.0 (9)
N7—Yb1—O1	69.99 (12)	C20—C21—H21A	109.5
O5—Yb1—N8	100.82 (13)	C20—C21—H21B	109.5
O2—Yb1—N8	26.03 (12)	H21A—C21—H21B	109.5
N1—Yb1—N8	68.10 (13)	C20—C21—H21C	109.5
O4—Yb1—N8	139.51 (12)	H21A—C21—H21C	109.5
N2^i^—Yb1—N8	151.76 (13)	H21B—C21—H21C	109.5
N6—Yb1—N8	92.51 (12)	C1—N1—Yb1	169.0 (4)
N5—Yb1—N8	95.38 (13)	C2—N2—Yb1^ii^	150.9 (3)
N7—Yb1—N8	78.34 (12)	C5—N5—C9	116.9 (4)
O1—Yb1—N8	25.75 (12)	C5—N5—Yb1	122.4 (3)
C1—Pt1—C2	92.89 (18)	C9—N5—Yb1	120.5 (3)
C1—Pt1—C3	178.77 (18)	C10—N6—C14	118.8 (4)
C2—Pt1—C3	86.46 (19)	C10—N6—Yb1	121.1 (3)
C1—Pt1—C4	88.27 (19)	C14—N6—Yb1	119.9 (3)
C2—Pt1—C4	178.60 (18)	C15—N7—C19	118.1 (4)
C3—Pt1—C4	92.37 (19)	C15—N7—Yb1	120.0 (3)
N1—C1—Pt1	178.7 (4)	C19—N7—Yb1	121.2 (3)
N2—C2—Pt1	172.2 (4)	O3—N8—O1	123.2 (5)
N3—C3—Pt1	174.7 (4)	O3—N8—O2	121.1 (4)
N4—C4—Pt1	177.5 (5)	O1—N8—O2	115.7 (4)
N5—C5—C6	123.6 (5)	O3—N8—Yb1	172.4 (4)
N5—C5—H5	118.2	O1—N8—Yb1	60.4 (2)
C6—C5—H5	118.2	O2—N8—Yb1	55.8 (2)
C7—C6—C5	118.7 (5)	N8—O1—Yb1	93.9 (3)
C7—C6—H6	120.7	N8—O2—Yb1	98.2 (3)
C5—C6—H6	120.7	Yb1—O4—H4A	122.0
C8—C7—C6	118.5 (5)	Yb1—O4—H4B	122.9
C8—C7—H7	120.8	H4A—O4—H4B	106.8
C6—C7—H7	120.8	Yb1—O5—H5A	122.5
C7—C8—C9	120.8 (5)	Yb1—O5—H5B	124.3
C7—C8—H8	119.6	H5A—O5—H5B	112.5

Symmetry codes: (i) *x*+1, *y*, *z*; (ii) *x*−1, *y*, *z*.

### Hydrogen-bond geometry (Å, °)

**Table:**

*D*—H···*A*	*D*—H	H···*A*	*D*···*A*	*D*—H···*A*
O4—H4A···N9	0.85	2.07	2.868 (7)	156.5
O4—H4B···N3^i^	0.85	2.28	3.125 (6)	169.6
O5—H5A···N3^iii^	0.85	1.96	2.802 (6)	170.4
O5—H5B···N4^iv^	0.85	2.00	2.842 (6)	172.7

Symmetry codes: (i) *x*+1, *y*, *z*; (iii) −*x*+1, −*y*+1, −*z*; (iv) −*x*+2, −*y*+1, −*z*.
